# Supplementary material for: Patterns of Intron Gain and Loss in Fungi
Source: PLoS Biol. 2004 Nov 30;2(12):e422. doi: 10.1371/journal.pbio.0020422 (PMC532390; doi:10.1371/journal.pbio.0020422)
Supplement: Table S1 — Also available at http://genes.mit.edu/NielsenEtAl/. (4.3 MB ZIP). [file pbio.0020422.st001.zip › NielsenEtAl/html/781.html]

AN0893.1.NCU09789.1.MG10691.1.FG05187.1


```
 CLUSTAL W (1.82) Multiple Sequence Alignments - Introns Inserted


Sequence 1: NCU09789.1	430 aa
Sequence 2: MG10691.1	423 aa
Sequence 3: FG05187.1	417 aa
Sequence 4: AN0893.1	424 aa
Alignment Length: 438 aa
Number Identitical Residues: 197 aa
Alignment Score (without introns) 10261


MG10691.1 	-MATIILGSQWG2DEG1KG~KLTDILCPSAEICARSA0GGHN~A~GHSIV~AQGVS~YDF
NCU09789.1	-MATLILGSQWG2DEG~KG1KLTDILCPKAQICARAA~GGHN~A~GHSIV1ANGVE~YDF
FG05187.1 	MTIKVILGAQWG1DEG0KG~KLTDILAPEAQLCARAA~GGHN~A1P---M~EYPTA1SSF
AN0893.1  	MAVTIVLGSQWG~DEG1KG~KITDMLSQEATLCCRAA~GGHN1A~GHTIV~HGNVT~YDF
          	   .::**:*** *** ** *:**:*. .* :*.*:* **** *   : :    .   .*

MG10691.1 	HLLPSGLVNPK------C~MNLIGSGVVFHV~PSFFSELEKLEAKGLAGVRDRIFVSDRC
NCU09789.1	HLLPSGLVNPN------C~MNLIGSSVVFHV~PSFFSELSKLEEKGLTDVHNRILVSDRC
FG05187.1 	HLLPSGLINPNWYYPKIC2LNFIGSGVVFHV2PSFFSEMAALIEKGLPSVEDRLLVSDRV
AN0893.1  	HILPSGLVSDK------C~INLIGAGTVVHV~PSFFKELAALEAKGLKGAAARAFISDRA
          	*:*****:. :      * :*:**:..*.** ****.*:  *  *** ..  * ::*** 

MG10691.1 	QVNFDLHAAVDGLEEVELGERKIGTTGRG~IGPSYSTKA0S~RSGVRISEVFDEAVFERK
NCU09789.1	HVNFDLHAAVDGLEEVELGDRKIGTTGRG~IGPSYSTKM~A0RSGVRIHEIFNEEIFERK
FG05187.1 	HIILQQHIAVDGLEEKELG---------G1IGPTYSKAS~A0REGVRLVDVFNKELFDSK
AN0893.1  	HVCFDLHSVVDGLEEKGLGGRKVGTTGKG~IGPCYSDKA~S~RRGVRVGEILDEEVFERK
          	:: :: * .******  **  . .::. * *** **    : * ***: ::::: :*: *

MG10691.1 	LRQLADGYRKRFGDLLKYDVEEEIARFK0EYRKLLPNYVVDAVKFIKDAQDQNRKILIEG
NCU09789.1	LRQLAAGYKKRFGDLLKYDVEEEIARFK~EYRVKLARYTVDAIQYMKEAQDRGYKILIEG
FG05187.1 	LRRLEAGYRKRFGDLFVYDVEEEIARFD~EYRPKLAKYAVDAVSFMKAAQEGNKNIIVEG
AN0893.1  	LRTLHAGYTARFG-QLDYDVEEEIARFK~EYRKLLVPHIVDQLAFFNKHKDSS-NTLVEG
          	** *  **  ***  : **********. ***  *  : ** : :::  :: . : ::**

MG10691.1 	ANALMLDIDYG1TYPYVTSSNPCLGGIITGLAINPRKIETIVGVVKAY0TTRVGDGIFKT
NCU09789.1	ANALMLDIDYG~TYPYVTSSNTGLGGIITGLAINPTKIDNIIGVVKAY0TTRVGGGPFKT
FG05187.1 	ANALMLDIKYG~SYPFVTSSNTTIGGIIGGLTLNPKNITETIGVVKAY~TTRVGQGAFKT
AN0893.1  	ANALLLDLDHG~TYPFVTSSSTGLGGAVQALHLNPTSIKSIIGVVKAY~TTRVGSGPFPS
          	****:**:.:* :**:****.. :** : .* :** .*   :****** ***** * * :

MG10691.1 	EDEGEIGTKLQDIGREWGVSTGRKRRCGWLDLVVVKYSA0AINHYTSLNLTKLDVLDTFP
NCU09789.1	EDLEEAGTKLQEIGREWGVSTGRKRRCGWLDLVVLKYST~AINNYTALNLTKLDILDTFE
FG05187.1 	EDTGEIGTKLQEIGREWGTSTGRRRRCGWLDLVVVKYSA~SINYYSALNLTKLDVLDTFE
AN0893.1  	EQLNEAGEKLQKVGREFGVTTGRKRRCGWFDLVLLRYSQ~SINHYTALNLTKLDILDDFD
          	*:  * * ***.:***:*.:***:*****:***:::**  :** *::*******:** * 

MG10691.1 	TLKVAVAYKDPATGEELDFFPADLSLLERCEVVYKEFEGWNTPTTHIKKF~EELPAQARQ
NCU09789.1	TIKVAVAYKDPQTGEEVEYFPADLDILDSLEVVYKELPGWNKPITDCKTY0YDLPKEARA
FG05187.1 	TIKIAIAYK--VDGEELDHYPADLDLLERAEVVYHEMPGWQKPTTGAKTF~YDLPKQARE
AN0893.1  	EIKVAVAYTLPDGTRLENTIPADAEVLNKVDVEYVTLPGWKSNTMGVKKY~EDLPENARK
          	 :*:*:**. .   .  :  *** .:*:  :* *  : **:.     *.:  :** :** 

MG10691.1 	YVE~FIEQYVG-VK~VGWIGTGPDREDMIYR-------
NCU09789.1	YIE~FIEEFVG-VP~ICYIGTGPKREDMIVRKTSAIKE
FG05187.1 	YIE0YIEKFIG-VK0VKWIGTGPDREAMIARA------
AN0893.1  	YIE~YIERELGGVP~VKWIGTGPARDDMICRE------
          	*:* :**. :*.*  : :***** *: ** *
```
